# SLC20A2-Associated Idiopathic Basal Ganglia Calcification-Related Recurrent Psychosis Response to Low-Dose Antipsychotics: A Case Report and Literature Review

**DOI:** 10.7759/cureus.12407

**Published:** 2020-12-31

**Authors:** Akito Uno, Hidetaka Tamune, Hisaka Kurita, Isao Hozumi, Naoki Yamamoto

**Affiliations:** 1 Department of Neuropsychiatry, Tokyo Metropolitan Tama Medical Center, Tokyo, JPN; 2 Department of Neuropsychiatry, Graduate School of Medicine, The University of Tokyo, Tokyo, JPN; 3 Neurology, Gifu Pharmaceutical University, Gifu, JPN

**Keywords:** idiopathic basal ganglia calcification (ibgc), fahr’s disease, antipsychotics, primary familial brain calcification (pfbc), risperidone, genotype-phenotype association, slc20a2, recurrent psychosis

## Abstract

Idiopathic basal ganglia calcification (IBGC), also known as Fahr’s disease or primary familial brain calcification, manifests as bilaterally symmetric calcifications in the brain. Clinical symptoms range from movement disorders to cognitive impairment and psychiatric symptoms. Since 2012, IBGC has been reported as an inherited disorder with several causative genes, including *SLC20A2*; however, the genotype-phenotype association remains unclear. Furthermore, longitudinal follow-up studies investigating the prognosis of neuropsychiatric symptoms in IBGC are lacking.

A 36-year-old woman who experienced recurrent psychosis since the age of 30 years was admitted to our hospital. Her symptoms included delusions, hallucinations, disorganized speech, and grossly disorganized behavior. Cranial CT revealed calcification of the bilateral basal ganglia and dentate nucleus. The possibility of metabolic or endocrinological disorders causing secondary calcification was excluded via laboratory examinations. The genetic analysis revealed *SLC20A2 *mutation, and therefore, she was diagnosed with definite IBGC. At the age of 37, 42, and 43 years, similar psychosis recurred due to a decrease in medication. Each episode was relieved within one week with a low dose of risperidone (1.5-2 mg/day p.o.). Eventually, remission was maintained with risperidone (1.5 mg/day).

To our knowledge, genetically confirmed case of IBGC with psychosis has been rarely reported. Recurrent psychosis can be the sole symptom of *SLC20A2*-associated IBGC and may be remitted with a low dose of risperidone. Literature review including eight case reports shows no superiority between medications. Although our case indicates that a low dose of antipsychotics can alleviate symptoms without any side effects and should be continued to prevent relapse in some patients with IBGC, there has been still shortage of the clinical evidence. Further longitudinal studies on genotype-phenotype associations may expedite personalized medicine for patients with IBGC.

## Introduction

Idiopathic basal ganglia calcification (IBGC), also known as Fahr’s disease or primary familial brain calcification, causes bilaterally symmetric calcifications in the brain, including the basal ganglia, dentate nucleus of cerebellum, thalamus, cerebral cortical gyrus, and deep cerebral white matter. Clinical manifestations range from motor symptoms to cognitive impairment and psychiatric symptoms. Exclusion of secondary factors such as endocrine disorders is necessary for diagnosis [1].

The pathophysiology of IBGC is mostly unknown, and the treatment is symptom-based. Although antipsychotics are used for treating psychosis, there is no standard regarding medication for psychosis in patients with IBGC. Causal genes for IBGC, such as* SLC20A2*, *PDGFRB*, *PDGFB*, *XPR1*, *MYORG*, and *JAM2*, have been reported since 2012 [2-7]; however, genotype-phenotype associations have not been elucidated. Additionally, there are limited reports on the psychiatric symptoms of patients with IBGC, especially with longitudinal clinical courses.

Here, we report a case of* SLC20A2*-associated IBGC with recurrent psychosis as the sole symptom in the 10-year observation period, which was treated with a low dose of risperidone.

## Case presentation

A 36-year-old woman was admitted to our hospital because of acute psychosis. She had no previous physical or family history, except that her father had mild cognitive impairment. Her mother had noticed the patient's transient bizarre behavior since she was 30 years old, including attempts to grasp empty space claiming, “I can collect the holy spirit.” One day before the first admission, she was in hallucinatory-delusional and psychomotor excitement states without daily fluctuation. On admission, she was treated with risperidone (2 mg/day p.o.) under the tentative diagnosis of schizophrenia based on Diagnostic and Statistical Manual of Mental Disorders, Fourth Edition, Text Revision (DSM-IV-TR). The hallucinatory-delusional symptoms improved remarkably within a few days. No apparent cognitive impairments, extrapyramidal symptoms, or cerebellar ataxic symptoms were observed throughout the admission; therefore, we excluded comorbid movement disorders.

**Figure 1:**
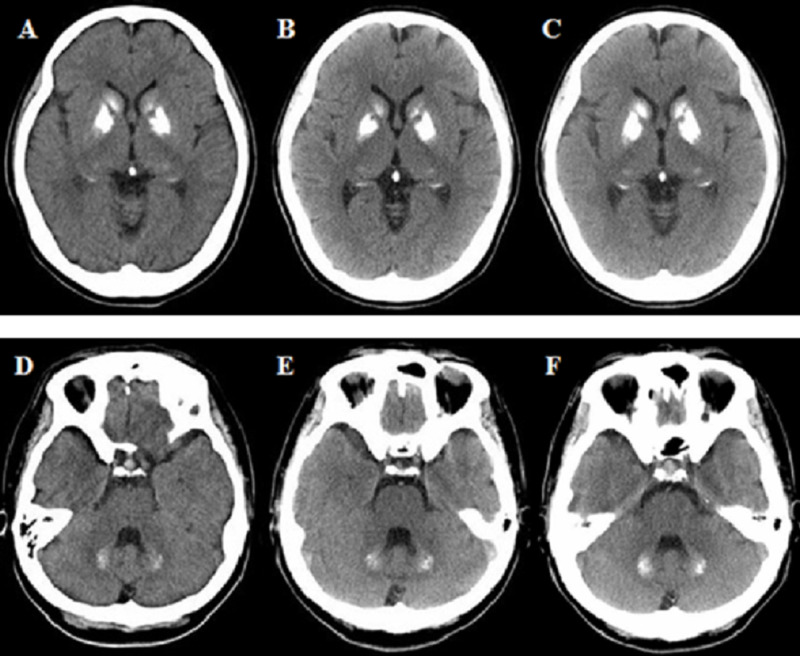
Longitudinal follow-up of the brain calcification in cranial CT Basal ganglia at the age of 37 years (A), 43 years (B), and 47 years (C); cerebellar dentate nuclei at the age of 37 years (D), 43 years (E), and 47 years (F).

Cranial CT revealed prominent calcification of the bilateral basal ganglia and cerebellar dentate nuclei (Figure 1). The laboratory examination data revealed that blood Ca^2+^, inorganic phosphorus (Pi), alkaline phosphatase, parathyroid hormone, somatomedin C, and insulin-like growth factor-binding protein-3 concentrations were all within the normal range. Hence, the possibility of metabolic or endocrinological disorders causing secondary calcification was ruled out.

Genetic analysis revealed a heterozygous SLC20A2 missense mutation (c.1487G > A, p.C496Y in exon 8) located at chromosome 8p11.21, which we published with its functional assessment elsewhere [8], confirming the clinical diagnosis of IBGC. The patient was discharged from the hospital within one month. After eight months, she arbitrarily terminated the medication and regular outpatient visits. After an additional five months, at the age of 37, she was involuntarily hospitalized as her psychosis returned three days prior to admission. The symptoms were relieved with risperidone (2 mg/day) after several days. She was discharged after one month and was maintained on risperidone (1 mg/day).

At the age of 42, her risperidone dose was decreased from 1 mg/day to 0.5 mg/day at her own discretion. After one month, she was involuntarily hospitalized due to disorganized behaviors such as suddenly taking off her mother’s dress; however, her symptoms improved within two days after increasing risperidone (1 mg/day), and she was discharged after two weeks. Later, the risperidone dose was increased to 1.5 mg/day, which relieved her anxiety during her hospital visits.

At the age of 43, two months after the risperidone dose was reduced to 1 mg/day on the patient’s request, she noticed signs of relapse and was voluntarily admitted to the hospital. The risperidone dose was increased to 1.5 mg/day, in which she recovered after a few days and was discharged after three weeks. During and after the hospitalization, her cognitive function was intact as her Mini-Mental State Examination (MMSE) score ranged from 28/30 to 30/30. Functional brain imaging with single-photon emission computed tomography (SPECT) and dopamine transporter (DAT) revealed no obvious signs of Parkinson’s disease or related disorders.

Further, with shared decision-making on requirement for maintenance treatment, she has remained on the same prescription (1.5 mg/day risperidone) with no relapse up to now (over four years). On clinical examination, no neurological findings including extrapyramidal or cerebellar symptoms, nor mood disorders, were noted for 10 years. Follow-up cranial CT revealed gradual but non-significant increases in the calcification of the bilateral basal ganglia and cerebellar dentate nucleus; the total calcification score [9] changed from 18 at 37 years to 20 at 47 years (Figure 1).

## Discussion

To the best of our knowledge, genetically confirmed IBGC case presenting only as recurrent psychosis has been rarely reported, and we have reported its longitudinal clinical course with a 10-year follow-up period. This case indicates that recurrent psychosis can be the sole symptom of *SLC20A2*-associated IBGC and can be maintained in remission with a low dose of antipsychotics.

*SLC20A2* is thought to be the most common causative gene of IBGC [10]. In a study on phenotypes of IBGC, out of 10 patients with *SLC20A2*-associated IBGC, five had cognitive impairment, five had psychiatric symptoms (psychosis [n = 1], mood disorders [n = 2], and others [n = 2]), five had movement symptoms, and three were asymptomatic [9]. A review reported mood disorders in IBGC had complex syndromes [11]; however, psychosis in IBGC is underrecognized and limited to some case reports (Table 1). Most of the reported cases have a short observational period and are complicated with cognitive impairment or movement disorders [12-18]. Only a few cases [19], including ours, have reported psychiatric symptoms alone.

**Table 1:** Summary of previously published IBGC case reports presenting with psychosis IBGC cases with psychosis due to a secondary cause were excluded. No published IBGC cases with psychosis reported the causal genetic mutation. B.G., Basal ganglia; D.N., dentate nuclei; EPS, extrapyramidal symptoms; N.A., not assessed; IBGC, idiopathic basal ganglia calcification.

	Sex	Age at onset	Psychiatric symptoms	Movement disorders	Cognitive impairment	Region of calcification	Medication per day
Shouyama et al., 2005 [12]	Female	31	Psychosis	EPS cerebellar sign	N.A.	B.G. D.N.	Haloperidol (8 mg) à risperidone (4 mg)
Nicolas et al., 2013 [19]	Female	39	Psychosis Depression	-	-	B.G.	Risperidone (2 mg) Venlafaxine (75 mg)
Pan et al., 2015 [13]	Male	41	Psychosis obsession	EPS (with olanzapine [10 mg])	-	B.G.	Olanzapine (10 mg à 2.5 mg) Fluoxetine (20 mg à 40 mg)
Younas et al., 2016 [14]	Male	44	Psychosis	EPS	+	B.G.	Donepezil (10 mg) à haloperidol (2 mg)
Mohapatra and Satapathy, 2016 [15]	Male	55	Psychosis	EPS	-	B.G.	Olanzapine (5 mg) Procyclidine (5 mg)
Naqvi et al., 2017 [16]	Male	18	Psychosis	Seizure	-	B.G.	Risperidone (4 mg) Procyclidine (10 mg)
Levina et al., 2019 [17]	Male	48	Psychosis	Romberg sign	N.A.	B.G. D.N.	Risperidone (3 mg)
Tololeski et al., 2019 [18]	Female	17	Psychosis	EPS pathological reflex	+	B.G. Thalamus	Quetiapine (900 mg) Sertraline (200 mg)
The present case	Female	30	Psychosis	-	-	B.G. D.N.	Risperidone (1.5 mg)

Classically, IBGC has been classified into two types of psychiatric symptoms: early-onset (median age: 30.7 years) and late-onset (median age: 49.4 years). Early-onset type causes psychosis and is rarely accompanied by movement disorders, whereas late-onset type is associated with dementia and movement disorders; however, these types have not been genetically defined [20]. Our present case is considered to belong to the early-onset type, and we hypothesized that a subgroup of patients with *SLC20A2*-associated IBGC may present recurrent psychosis.

As our present case indicated, a low dose of antipsychotics can alleviate the symptoms of patients with IBGC without any side effects and should be continued to maintain remission, especially in patients with *SLC20A2*-associated IBGC. Since no established guidelines are available, medications for psychosis in IBGC patients should be prescribed while considering side effects such as neuroleptic malignant syndrome and extrapyramidal symptoms. Studies have reported the use of risperidone, olanzapine, quetiapine, and haloperidol for psychosis, with relatively small dosages (Table 1). In the present case, risperidone (1.5 mg/day p.o.) maintained remission, and its withdrawal resulted in relapses, thus indicating that the dose was necessary and sufficient. The optimal dose would need to be adjusted for each patient.

Pathophysiologically, *SLC20A2* gene encodes PiT-2, which is a type III Na-dependent Pi transporter. Pi level is reported to be elevated in the cerebrospinal fluid of patients with IBGC [8]. Further, it is postulated that the cell calcification mechanism involves the complex formation between extracellular Pi (high concentrations) and Ca^2+^ and other metal ions, which causes Ca^2+^ ions to flow into cells and promote bone differentiation; however, no relationship between the gene mutation site and degree of calcification or clinical phenotype has been established, partly because of insufficient information on the phenotype [1,9]. As in our case, longitudinal studies on the genotype-phenotype association are warranted to understand the pathophysiology of IBGC, enable personalized medicine, and predict prognosis, including recurrent clinical course.

## Conclusions

Recurrent psychosis can be the sole symptom of *SLC20A2*-associated IBGC and can be maintained in remission with a low dose of antipsychotics. Literature review including eight case reports shows no superiority between medications. Although our case indicates that a low dose of antipsychotics can alleviate symptoms without any side effects and should be continued to prevent relapse in some patients with IBGC, there has been still shortage of the clinical evidence. Further longitudinal studies on genotype-phenotype associations may expedite personalized medicine for patients with IBGC.
